# Anti-Cancer Stem-Cell-Targeted Therapies in Prostate Cancer

**DOI:** 10.3390/cancers15051621

**Published:** 2023-03-06

**Authors:** Samantha Gogola, Michael Rejzer, Hisham F. Bahmad, Ferial Alloush, Yumna Omarzai, Robert Poppiti

**Affiliations:** 1 Department of Translational Medicine, Herbert Wertheim College of Medicine, Florida International University, Miami, FL 33199, USA; 2 The Arkadi M. Rywlin M.D. Department of Pathology and Laboratory Medicine, Mount Sinai Medical Center, Miami Beach, FL 33140, USA

**Keywords:** prostate cancer, therapy resistance, cancer stem cells, targeted therapy

## Abstract

**Simple Summary:**

The standard of care therapy for early prostate cancer (PCa) includes external beam radiation therapy (EBRT), brachytherapy, radical prostatectomy, active surveillance, or a combination approach. For advanced disease, androgen deprivation therapy (ADT) and other neoadjuvant therapies are considered. Nevertheless, castration-resistant prostate cancer (CRPC) develops in many patients. This instigated the development of novel therapeutic approaches using targeted therapies, including prostate cancer stem cell (PCSC)-targeted therapies. Here, we summarize the mechanisms of action of PCSC-targeted therapies and discuss avenues of future development.

**Abstract:**

Prostate cancer (PCa) is the second-most commonly diagnosed cancer in men around the world. It is treated using a risk stratification approach in accordance with the National Comprehensive Cancer Network (NCCN) in the United States. The main treatment options for early PCa include external beam radiation therapy (EBRT), brachytherapy, radical prostatectomy, active surveillance, or a combination approach. In those with advanced disease, androgen deprivation therapy (ADT) is considered as a first-line therapy. However, the majority of cases eventually progress while receiving ADT, leading to castration-resistant prostate cancer (CRPC). The near inevitable progression to CRPC has spurred the recent development of many novel medical treatments using targeted therapies. In this review, we outline the current landscape of stem-cell-targeted therapies for PCa, summarize their mechanisms of action, and discuss avenues of future development.

## 1. Introduction

### 1.1. Epidemiology

Prostate cancer (PCa) is the second-most common cancer in men around the world, with 288,300 estimated new cases and 34,700 estimated deaths in the United States in 2023 [1,2]. In the United States alone, 13 out of every 100 men will be diagnosed with PCa in their lifetime [3]. Surveillance, epidemiology, and end results (SEER) data concluded that between 2010 and 2015, the overall incidence of PCa in men decreased for low-risk disease. However, the incidence of high-risk disease, including metastasis, increased from 6.2 to 7.1 in men aged 50 to 74 years and from 16.8 to 22.6 in men greater than the age of 75 years [3]. The decreased incidence in low-risk disease can at least partly be due to the 2012 change from the United States Preventive Services Task Force (USPSTF) [4], which concluded that PCa screening using prostate-specific antigen (PSA) should not be recommended [4]. In 2018, the USPSTF recommendation changed again, recommending a personalized approach for PCa screening in men aged 55 to 69, which may again be associated with a change in both screening practices and the apparent incidence of disease [5].

### 1.2. Screening and Diagnosis

Without proper screening, many cases of PCa may remain clinically silent [5,6,7]. Carcinogenesis often occurs so slowly that most men with PCa die from other unrelated causes before the disease becomes advanced enough to be detected [6,7,8]. This fact underscores the ongoing debate concerning the usefulness of PCa screening and likely accounts for the continuously changing USPSTF guidelines. For disease screening to be useful, it must be effective at reducing disease-specific morbidity and mortality by detecting the disease at an early stage. In prostate cancer, early-stage detection does not necessarily correlate with a decrease in morbidity and mortality nor a clinically beneficial outcome for the patient.

Screening increases the detection of PCa among men and thus increases the apparent incidence. For example, the development of prostate-specific antigen (PSA) testing was followed by a sharp increase in the reported incidence of PCa, but has since returned closer to original levels as PSA testing has declined [9]. According to a recent meta-analysis, PSA testing was found to effectively aid in diagnosing more men with PCa than in those who were not screened at all [10]. This screening was also shown to reduce the risk for late-stage PCa, with the absolute risk reduction of high-grade metastatic disease being 3.1 per 1000 men as found by the European Randomized Study of Screening for Prostate Cancer (ERSPC) [11].

PCa mortality rates have significantly decreased since the development of PSA testing; however, it is unclear if this decrease is due solely to PSA testing specifically or there are other contributing factors [10,11]. Another important consideration with more screening is the increased risk of false-positive results [12,13,14]. A prostate biopsy is the next diagnostic step following a sufficiently elevated PSA, meaning that a greater number of men may be subjected to the stress of unnecessary testing and treatment with its potential related morbidities [15,16]. For example, for every 1000 men screened for PCa using PSA, 1 will need hospitalization for sepsis, 3 will need pads for urinary incontinence, and 25 will develop erectile dysfunction [10]. As such, it is imperative to continue collecting data and adjust recommendations as needed.

### 1.3. Grading of Prostate Cancer

The most common type of carcinoma arising in the prostate is adenocarcinoma. Upon diagnosis, the tumor is graded using the Gleason scoring system, which correlates with the patient’s overall prognosis and, on biopsy specimens, helps guide treatment and management protocols. Gleason grading relies on the identification of architectural growth patterns of the tumor [17,18]. It is divided into five patterns with Gleason grade 1 being the most (well) differentiated and Gleason grade 5 being the least (poorly) differentiated [19]. A known caveat of the Gleason scoring system is that grades 1 and 2 are unable to be reliably identified in histology and therefore are not diagnosed in practice or applied in the Gleason scoring system.

**Figure 1:**
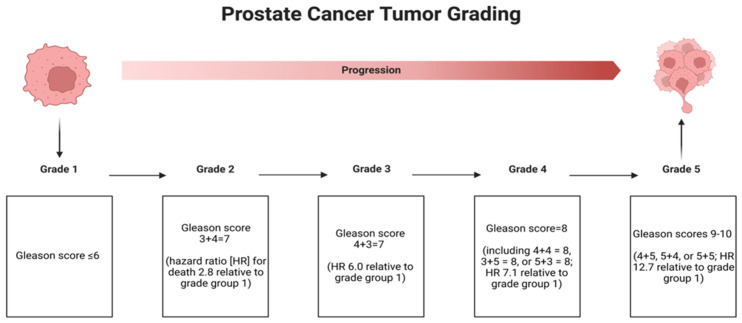
PCa tumor grading using the Gleason and ISUP grading system. Created with BioRender.com (2023).

The Gleason score represents a composite of the two most prevalent Gleason grade patterns identified [20]. The most predominant (primary) Gleason grade is numerically added to the next most predominant (secondary) Gleason grade, and the sum is known as the Gleason score, which ranges from 6 to 10 [20]. The higher the Gleason score, the greater the risk of having metastatic disease as well as a worse clinical outcome following treatment of localized disease [17,18]. Recently, the International Society of Urologic Pathology (ISUP) introduced a newly devised a Grade Group designation, which correlates each Gleason score with one of five distinct successive categories known as the Grade Group [20,21]. This has been shown to provide more accurate stratification than the Gleason core alone. The Gleason Score and Grade Group are reported in tandem [20,21] (Figure 1).

### 1.4. Treatment of Prostate Cancer

PCa is treated using a risk-stratification approach in accordance with the National Comprehensive Cancer Network (NCCN) [22]. The main treatment options for early PCa include external beam radiation therapy (EBRT), brachytherapy, radical prostatectomy, active surveillance, or a combination of these. In those with advanced disease, androgen deprivation therapy (ADT) is considered first-line therapy. ADT is used in patients with advanced metastatic disease and can be used alone or in combination with chemotherapy [23,24]. The approach to this therapy is through bilateral orchiectomy or medical castration using a gonadotropin-releasing hormone (GnRH) agonist, which may be used alone or along with androgen blockade [23,24].

### 1.5. Mechanisms of Progression into Castration-Resistant Prostate Cancer

ADT is used in patients with advanced PCa [25]. During androgen-dependent progression, PCa cells largely rely on the androgen receptor (AR) for both growth and survival [26,27,28]. Testosterone enters the cell by simple diffusion and is converted to dihydrotestosterone (DHT) using the cytoplasmic enzyme 5-alpha-reductase [26]. DHT has a five-to-ten-fold increased affinity for the AR compared to testosterone [26]. When DHT binds to the receptors in the cytoplasm, the AR undergoes phosphorylation, dimerization, and translocation into the nucleus, thus binding to the androgen-response elements within the host DNA and triggering transcription of genes involved in growth and survival [26].

Despite ADT, the vast majority of PCa will eventually progress to a disease state called castration-resistant prostate cancer (CRPC) [29]. The transition from androgen dependent to androgen independent tumors is still not well-understood, although continuous AR signaling in the absence of circulating androgens and AR blockage appear to be central factors [26,27,28,29,30]. Additional hypothesized mechanisms include AR gene amplification, AR gene mutations, ligand-independent activation of the AR, involvement of coregulators, and recruitment of tumor stem cells [26,27,28,29,30].

### 1.6. Need for Targeted Therapies

The near inevitable progression to CRPC and lack of effective treatment options has illustrated the significant need for novel therapies. While many pathways involved in the development of CRPC are still unknown, continued investigation into developmental and potentially oncogenic pathways have elicited the discovery of new and promising targeted therapies [29,31,32,33]. These novel therapies are being approached by either specifically targeting the critical pathways or targets or through multi-targeted therapies that affect both the cancer cell and the surrounding microenvironment of the tumor [29,31,32,33]. While progress is being made toward elucidating the critical components of CRPC progression, significant work is still needed in identifying and testing potential new treatments [29].

## 2. Cancer Stem Cells in Prostate Cancer

A very small proportion of cells that are CD44^+^/α_2_β_1_/CD133^+^ and do not express AR, comprising less than 0.1 percent of the tumor volume, have been identified as prostate cancer stem cells (PCSC) [34,35]; the hierarchy stem cell model of PCa states that only this small subset of cells is responsible for the androgen-independent cell growth seen in PCa [35,36]. Since these cells are androgen-independent in nature, they are free to continue to grow in an androgen-depleted environment and continue to multiply despite receiving ADT [35,36]. These cancer stem cells (CSC) differentiate into both androgen-dependent and androgen-independent cells, accounting for the heterogenous androgen phenotype that is often observed in CRPC patients [15,37]. This mechanism of tumor growth indicates the need for specific stem cell therapies [35]. Conventional therapies eradicate the majority of cells within the tumor, but they offer only a temporary solution to a growing problem if the residual PCSCs are able to continue proliferating [35].

## 3. Targeting Prostate CSC-Related Signaling Pathways

### 3.1. Hedgehog (Hh) Pathway

**Figure 2:**
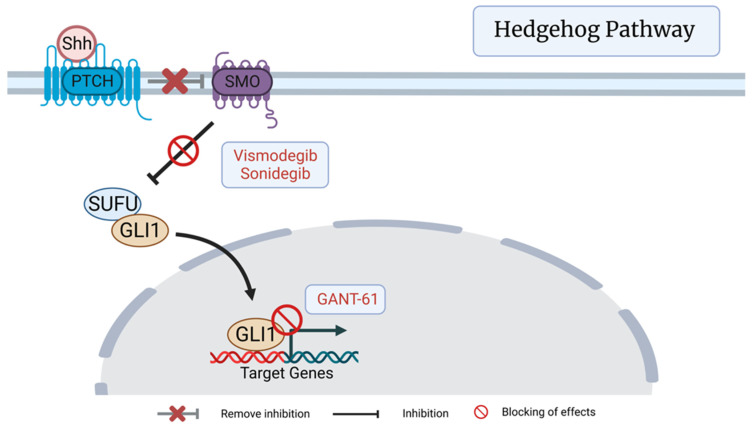
Hedgehog signaling pathway featuring the SMO antagonists Vismodegib and Sonidegib and Gli antagonist GANT-61. Created with BioRender.com (2023).

The hedgehog (Hh) signaling pathway is an important orchestrator of development in the embryonic prostate and epithelial regeneration in the adult prostate [38]. The pathway begins with a secretory Hh ligand binding to the transmembrane Patched (Ptc) receptor, which removes the inhibitory action of the Smoothened (Smo) receptor and allows *Gli* transcription factors to translocate to the nucleus and promote cell differentiation [39]. Increased activity of this pathway has been implicated in metastatic progression of PCa, opening several potential avenues for targeted therapies [40]. Initial efforts have primarily focused on blocking the pathway intermediate Smo due to its membrane accessibility, but therapy resistance has prompted additional work on downstream effector targeting. The Smo-antagonists CDC-0449 (Vismodegib) and Sonidegib showed early promise with treating basal cell carcinoma but less robust results when utilized for PCa [41,42,43]. It has been postulated that both downstream oncogenic activation pathways and an absence of primary cilium, the site of *Gli* activation by Smo, in PCa cells has thus far blunted results with direct Smo targeting [44]. However, the downstream Gli-antagonist *GANT-61* has demonstrated a more robust impact on suppressing PCa stem cell survival and self-renewal [44]. Of note, Gonnissen et al. observed an increased radiosensitivity in *GANT-61*-treated PCa xenografts [45]. In vitro, cell-intrinsic sensitivity was mediated by *GANT-61* inhibition of *Gli1* resulting in downstream activation of p53 signaling and cell cycle arrest with apoptosis [45]. In vivo, additional radiosensitization was hypothesized to come from inhibition of Hh signaling in the surrounding tumor stroma, a known contributor to tumorigenesis [46]. Several subsequent studies have shown promise with *GANT-61* promoting increased sensitization to other molecular pathway targeting drugs, further emphasizing the need for additional research into relevant pathway interactions and therapeutic applicability [46,47] (Figure 2).

### 3.2. Wnt Signaling Pathway

**Figure 3:**
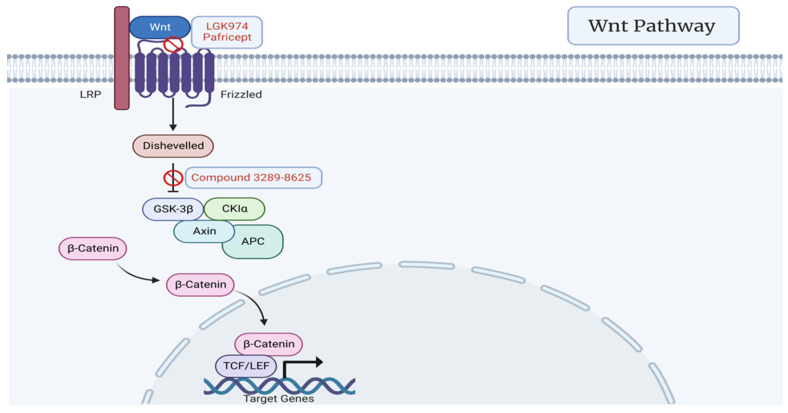
Wnt signaling pathway featuring Porcn inhibitor LGK974, WNT receptor decoy Pafricept, and Dvl signaling inhibitor compound 3289-8625. Created with BioRender.com (2023).

The Wnt signal cascade plays a critical role in cell fate determination, embryonic patterning, cell proliferation, survival, and differentiation [48]. The pathway begins with a specific Wnt ligand binding its specific Frizzled (Frz) transmembrane receptor, leading to activation of the Dishevelled (Dvl) protein that frees β-catenin, a protein that mediates cell proliferation and differentiation [49]. Wnt signaling is often activated in PCa and has been correlated with progression to CRPC, higher Gleason scores, elevated PSA levels, earlier disease onset, and higher rates of recurrence [50,51,52,53,54]. As such, a number of inhibitor molecules have been developed to exploit this important pathway through Wnt inhibitory factors, Wnt antagonists, or conditional knockout of β-catenin [55,56,57]. Compound 3289-8625 inhibits signaling at the level of Dvl and has been shown to inhibit the growth of PC3 Pca cells in vitro [58]. LGK974 is an inhibitor of the Porcupine (Porcn) enzyme responsible for the palmitoylation of Wnt prior to Frz binding, which is a crucial step in the process of Wnt ligand secretion [59]. Foxy-5 is a WNT5A-mimicking peptide that specifically impairs prostate cancer invasion by inhibiting endothelial tumor cell migration through activation of Wnt-5a-mediated signaling [60]. Pafricept (OMP-54F28) is a WNT receptor decoy and fusion protein of FZD8 ligand-binding domain that binds to all Wnt ligands [61] (Figure 3).

### 3.3. Notch Pathway

**Figure 4:**
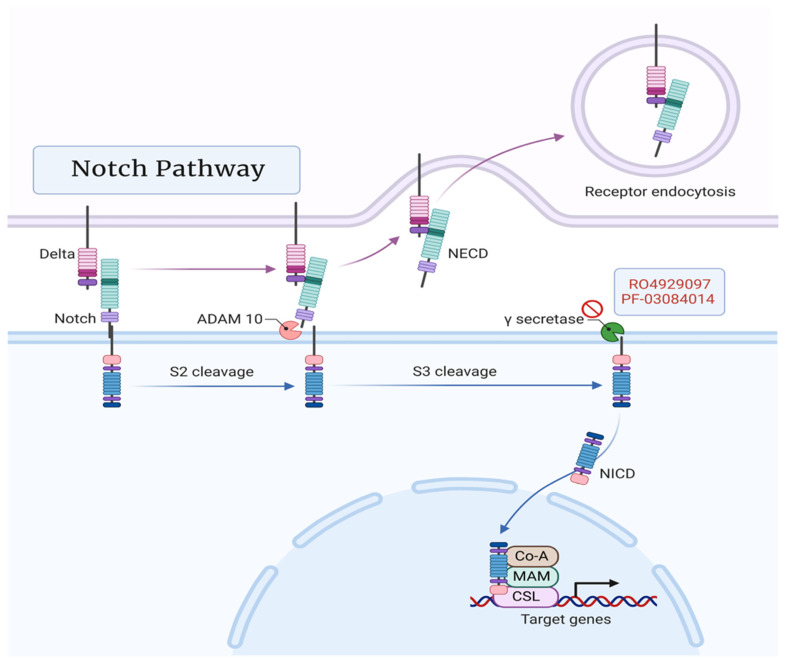
Notch signaling pathway featuring GSIs RO4929097 and PF-03084014. Created with BioRender.com (2023).

Notch glycoproteins are a family of transmembrane cell surface receptors that participate in the transmission of growth and proliferation signals [62]. Upon ligand activation, a series of cleavage reactions leads to activation of the gamma secretase complex, which ultimately releases a Notch intracellular domain [63]. This activated Notch translocates to the nucleus to act as a transcriptional co-activator of genes for progenitor cell differentiation and pluripotent stem cell self-renewal [62]. As such, an increase in Notch signaling promotes tumor cell proliferation by maintaining tumor cells in a stem-cell-like proliferative fate [64]. It has previously been demonstrated that an upregulation of Notch signaling plays a role in PCa epithelial-to-mesenchymal transition, metastasis, and progression to CRPC [65,66,67,68]. Similarly, multiple inhibitory strategies targeting the Notch pathway have produced growth suppression, apoptosis, and increased sensitivity to cytotoxic chemotherapy [69,70,71]. Gamma-secretase inhibitors (GSI) have produced some promising results in various cancers; however, RO4929097 is the only GSI trialed in Pca to date [72]. RO4929097 demonstrated an initial preclinical and Phase 1 ability to produce slower-growing tumor phenotypes with good tolerance [73]. A Phase 2 clinical trial sought to exploit this propensity to delay re-growth following anti-androgen therapy, but the trial was ended early due to a lack of available drug [74]. Of note, recent studies with the GSI PF-03084014 have shown promising preclinical results in decreasing tumor growth with or without docetaxel in both Pca and CRPC, warranting further investigation [75,76] (Figure 4).

### 3.4. NF-κB Signaling Pathway

**Figure 5:**
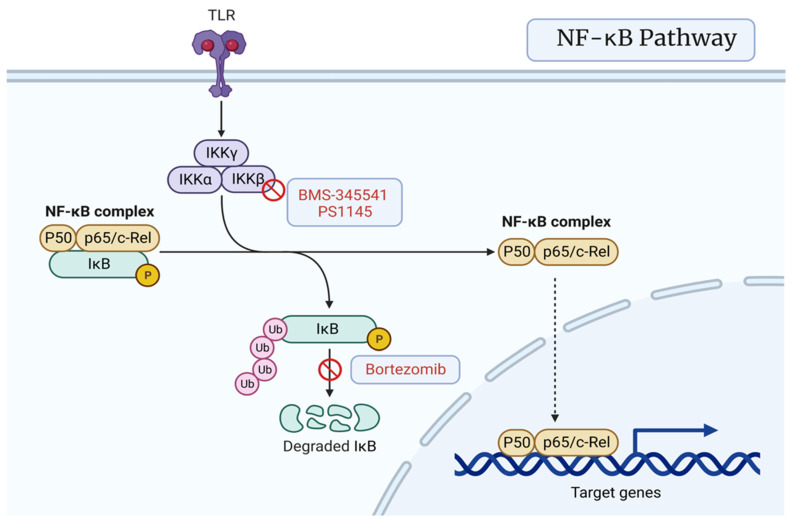
NF-kB signaling pathway featuring IkB degradation inhibitor bortezomib and IKK-beta inhibitors BMS-345541 and PS1145. Created with BioRender.com (2023).

Nuclear factor kB (NF-kB) plays a major role in apoptosis by regulating the transcription of *Bcl-2* [77]. Activation of the NF-kB pathway in PCa cells leads to Pca progression, metastasis, recurrence, and resistance [78,79]. This implies that inhibiting signaling can combat antitumor responses while increasing the vulnerability of tumor cells to anticancer medications [80]. Bortezomib is a current anti-tumor medication on the market that utilizes proteasome inhibitors to inhibit IKB-alpha degradation through inhibition of the ER-associated protein degradation (ERAD) mechanism, which involves the retrograde translocation or dislocation of misfolded proteins out of the ER and subsequent degradation by cytosolic 26S proteasomes [80]. It has an overall inhibitory effect on NF-kB signaling and is able to inhibit cell growth and cause apoptosis in many different cancer cell lines [80]. It can also help in overcoming drug resistance when combined with conventional therapeutic agents or radiation and has shown anti-tumor activity when used alone in those with advanced CRPC [80]. One method of tumor proliferation is through the independent activation of inhibitor of kB kinase (IKK) [80]. BMS-345541 and PS1145 are novel compounds that are highly selective for IKK-beta, leading to inhibitory activity, thus triggering cell apoptosis in androgen receptor-expressing Pca cell lines [81]. BKM120 acts downstream of the NF-kB pathway as a strong and highly selective pan-class I PI3K inhibitor [82]. 17-(allylamino)-17-demethoxygeldanamycin is a heat-shock protein 90 (HSP90) inhibitor [83]. Aspirin is being evaluated as a mechanism for targeting this pathway, since inflammation may have a key role in the progression of prostate cancer through unknown mechanisms, and aspirin has been shown to prevent several inflammation-related tumors [84] (Figure 5).

### 3.5. PI3K/AKT/mTOR Pathway

**Figure 6:**
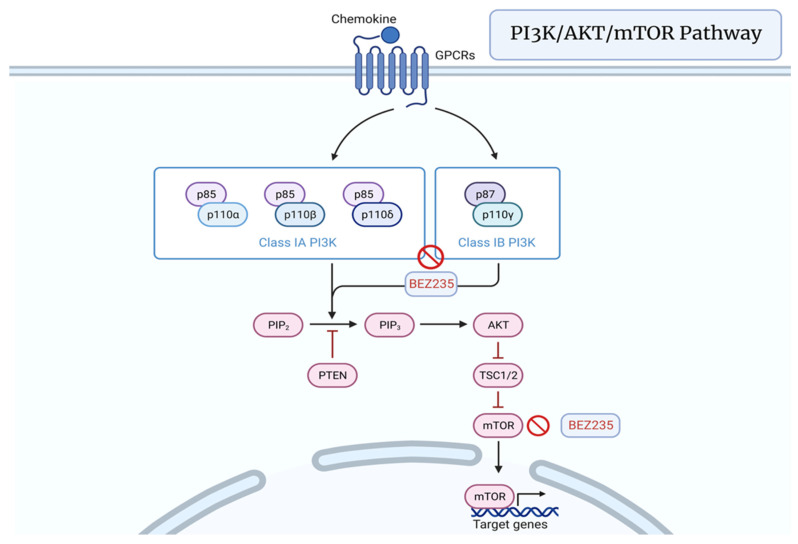
PI3K/AKT/mTOR signaling pathway featuring the PI3K/mTOR inhibitor BEZ235. Created with BioRender.com (2023).

Alterations in the PI3K/AKT/mTOR pathway have been associated with a variety of malignancies due to its well-documented involvement with development, cell growth, proliferation, malignant transformation, metastasis, tumor progression, apoptosis, and resistance [85]. It has been found that the signaling of this pathway is up-regulated in 30 to 50 percent of PCa subjects with phosphatase and tensin homolog (PTEN) suppression or inappropriate activation of both AKT and S6 [86]. In prostate epithelial cells, suppression of PTEN or increased expression of AKT resulted in PI3K/AKT/mTOR activation sufficient for development of in vivo PCa [87]. BEZ235 is a dual inhibitor of PI3K and mTOR that has been shown to reduce the tumor volume in PCa, which was mediated by the loss of PTEN [88] (Figure 6).

## 4. Targeting Prostate CSC Microenvironment

The prostate CSC niche is a microenvironment for stem cells that maintains a stem-like state [89,90,91,92]. CSCs rely on a similar niche, which is able to control the differentiation and self-renewal of these cells [90]. The CSC niche provides a microenvironment that regulates the balance between quiescence and self-renewal, actively responding to any requirements of homeostasis [90]. This has been supported through the finding that loss of the CSC niche results in the loss of CSCs [91]. The CSC microenvironment has been shown to protect CSCs from drug-induced apoptosis and leads to resistance [90,93]. Furthermore, it has been implicated with abnormal induction of Hh, Wnt, NF-κB, Notch, PI3K/AKT/mTOR, and TGF-beta pathways and is directly involved in the development of metastasis [94,95,96]. Bevacizumab is a monoclonal antibody that can be used to target vascular endothelial growth factor (VEGF) to disrupt the CSC microenvironment through the reduction of neovasculature at the level of the stromal epithelium [97]. However, it was found that many PCSCs themselves are resistant to bevacizumab through Rac1-mediated ERK activation, with subsequent studies showing that inhibition of Rac1 and downregulation of P-Rex1 increased the sensitivity of these cells to bevacizumab [97].

## 5. Immunotherapies Targeting Prostate CSCs

### 5.1. (CAR)-Modified T-Cell Therapy Targeting CSC-Associated Tumor Antigens

PCSCs have been shown to have increased expression of various cell surface markers with potential for immunotherapeutic targeting [98]. Chimeric antigen receptor T-cell (CAR-T) therapy is a relatively recent treatment approach that exploits this differential expression and has shown great promise with both hematologic malignancies and solid tumors [99,100,101]. By engineering T cells with artificial receptors specific for pre-selected tumor associated antigens (TAA), CAR-T has the potential for highly precise and efficacious targeting of CSCs [102].

### 5.2. Anti-CD133 CAR-T Therapy

CD133 (prominin-1) is a well-documented CSC biomarker in a number of solid tumors and represents an interesting potential target for PCSC prognostication and therapeutic intervention [103]. Collins et al. originally described the isolation of CD44+/α2β1high/CD133+ PCSCs and their ability to reconstitute cancer bulk on xenograft inoculation [35]. Subsequently, a gene profiling analysis of CD133+ cells performed by Kanwal et al. identified an array of upregulated stem cell markers consistent with enhanced clonogenic and tumorigenic capacity [104]. Initial efforts at CAR-T targeting of CD133 have shown promising results in a number of solid tumors. Zhu et al. derived AC133-CAR-T cells for glioblastoma multiforme that demonstrated good CSC recognition and inhibition of orthotopic xenograft growth [105]. Additionally, a phase 1 clinical trial of CD133-CAR-T cells from Wang et al. further supported the feasibility, controllable toxicities, and effectiveness of CD133-targeted therapy for a number of highly metastatic malignancies [106]. Studies investigating CD133-CAR-T therapy specifically for PCSCs remain outstanding, but the growing body of evidence linking CD133 activity to PCSC stemness supports further investigation into this immunotherapeutic approach.

### 5.3. CAR T Cells Targeting the CSC Marker EpCAM

Epithelial cell adhesion molecule (EpCAM) is another well-known biomarker commonly overexpressed in PCSCs [107]. Multiple studies have demonstrated a positive association between the degree of overexpression and the progression of PCa metastasis and resistance [108,109]. An early preclinical investigation by Deng et al. showed early promise with significant tumor-killing ability of EpCAM-CAR-transduced human peripheral blood lymphocytes both in vitro and in vivo [110]. However, the distribution of EpCAM in normal epithelia has raised some concerns for off-tumor immunopathology, particularly regarding adverse pulmonary effects [111]. Only a single clinical trial with an unknown recruitment status has been initiated to investigate EpCAM-CAR-T therapy against PCSCs. As such, additional studies are needed to further determine the efficacy and toxicity of EpCAM-targeted interventions in PC.

## 6. Targeted Nanoparticles

The use of nanoparticles as chemotherapeutic delivery vehicles is a developing avenue of targeted tumor therapy. Originally, nanomedicine relied on the increased penetrability of tumor vasculature; however, more recently developed nanoparticles exploit tumor surface moieties for more precise targeting [112,113]. PCSC, commonly identified by their CD44, α2β1, and CD133 surface markers, appear to be ideal candidates for this treatment modality. Traditional drugs developed for PCa, including abiraterone acetate, cabozantinib, and docetaxel, have inherent drawbacks in PCSC populations with relatively poor targeting, tumor resistance, and a variety of adverse effects [114,115,116]. Nanomedicine therapies offer the potential to avoid these pitfalls through the highly specific targeting of PCSC populations. Thus, with their enhanced utilization efficiency and reduced toxicity profile, nanoparticle interventions represent an enticing direction for future therapeutic innovation [117].

### 6.1. CD44-Targeting Therapies

CD-44 is a transmembrane glycoprotein commonly used in the identification of PCSC, with the overexpression of CD44 being associated with increased PCa tumorigenicity and metastasis [118]. To date, a variety of nanotherapeutic approaches have been developed that tend to either utilize hyaluronic acid (HA), the primary ligand of CD44, or CD44 antibodies for drug targeting. Huang et al. developed a HA-based nanoparticle to deliver bioactive epigallocatechin-3-gallate to CD44-positive PCa cells [119]. In vitro results demonstrated efficient nanoparticle internalization via ligand–receptor recognition with inducible G2/M phase cell cycle arrest and inhibition of PCa cell growth [119]. Subsequent in vivo results confirmed the specificity of CD44 binding, as well as the nanoparticle’s ability to promote PCa cell apoptosis and significantly decrease tumor activity and tissue inflammation [119]. In a different approach, Wei et al. generated nanoparticles with CD44 antibodies (SM-LPN-CD44) to deliver the potent therapeutic agent salinomycin to CD44-positive PCSC [120]. In vivo assays demonstrated an enhanced ability for the SM-LPN-CD44 nanoparticles to deliver salinomycin to PCSC compared to non-targeted nanoparticles, with the SM-LPN-CD44 nanoparticles also showing a significant decrease in CD44-positive cells and drug concentration needed to inhibit 50% of tumor growth (IC50) [120]. Mahira et al. opted to develop a variant of the HA-targeted method by producing cationic liposomes coated with HA to deliver the chemotherapeutic agents cabazitaxel and silibinin directly to CD44-positive PCSC [121]. Their results demonstrated that the HA-coated liposomes had significantly increased cytotoxicity in the target cells, with a lower IC50 and improved colony formation inhibition, G2/M phase arrest, and induction of apoptosis [121]. Sanfilippo et al. sought to employ another variant of the HA-targeting model by utilizing synthesized spherical gold nanoparticles capped with low and high molecular weight HA [122]. The results showed a higher rate of CD44-positive PCa cellular uptake for nanoparticles functionalized with HA compared to those without HA, supporting this approach as another possible targeting system for therapeutic delivery [122]. In a more recent study, Pramanik et al. explored yet another variant of the HA-targeting method by creating HA-functionalized liquid crystalline lipid nanoparticles, termed cubosomes, to deliver chemotherapeutic copper acetylacetonate to CD44-expressing tumors [123]. Their results showed increased selective uptake of the targeted-cubosomes by CD44-positive cells compared to non-targeted-cubosomes, as well as significantly elevated rates of apoptotic cell death induced by the targeted cubosomes [123]. While this study only utilized breast and colon cancer cell lines, the selective CD44-targeting of the nanoparticle shows potential for use in other solid tumors, such as PCa, and warrants further investigation.

### 6.2. CD133-Targeted Therapy

CD133 is a transmembrane glycoprotein thought to help organize cell membrane topography and is the most frequently used cell surface marker to detect and isolate cancer stem cells from a variety of solid tumors, including PCa [124]. There are a number of studies that have shown promising early results in targeting CD133 with antibodies or oligonucleotides known as aptamers. Tan et al. utilized a CD133-antibody approach by functionalizing a gold nanoprobe (GNS@IR820/DTX-CD133) to deliver IR820 and docetaxel to PCa cells for synergistic photothermal therapy, photodynamic therapy, and chemotherapy [125]. In vivo results demonstrated a significantly higher cellular uptake efficiency and number of dead PCa cells with the targeted nanoprobe compared to non-targeted interventions, supporting the importance of CD133-targeting in therapeutic efficacy [125]. In the realm of aptamer targeting, Ma et al. developed curcumin-containing lysosomes embedded with the aptamer A15, which has shown promise in specifically targeting CD133-positive cancer stem cells [126,127]. Their results showed significantly higher rates of PCa-specific drug internalization, inhibition of cell growth, and decreased tumor volume in the A15-targeted group compared to the non-targeted groups [127]. As a whole, each of these preclinical studies highlight the potential for CD133-mediated PCSC therapeutic targeting and support the need for additional investigation.

## 7. Anti-CSC Targeted Therapies: Clinical Trials

More than 50 clinical trials were identified on www.clinicaltrials.gov (accessed on 15 January 2023) to involve PCa and one of the previously discussed targeted therapies. Several of these were excluded from further review due to their only being indirectly involved with PCSCs. For example, study NCT03103152 involving aspirin measured the rate of patient recruitment to a randomized chemoprevention study with a secondary outcome of measuring response to treatment in those receiving aspirin and/or D3 via MRI. Study NCT00349557 is working to determine the acute toxicities from IMRT with other therapies including bevacizumab. Study NCT03878524 is looking at the feasibility of implementing an individualized treatment strategy in those receiving SMMART-PRIME therapy including bevacizumab. Study NCT03878524 is investigating the feasibility of implementing individualized therapy with GDC-0449. Study NCT03878524 is looking at the feasibility of implementing an individualized treatment strategy with bortezomib. Because of these exclusions, 52 of these clinical trials were investigated further. The status of each trial is listed as recruiting, completed, terminated, or unknown based on its latest update. The overall profile of each targeted therapy is summarized below with a full listing of included clinical trials available in Table 1.

### 7.1. Hedgehog (Hh) Pathway

One completed clinical trial (NCT02111187) involving Soniedegib found that six of seven participants had at least a twofold reduction in *GLI1* expression following treatment when comparing one group that received Sonidegib prior to prostatectomy and another that underwent prostatectomy alone.

### 7.2. WNT Signaling Pathway

Awaiting the results for two clinical trials (NCT0202029, NCT02655952) investigating the safety profiles of Foxy-5.

### 7.3. Notch Pathway

A clinical trial (NCT01200810) for RO4929097 was terminated due to a lack of available drug.

### 7.4. NF-kB Pathway

Following treatment with a combination of weekly docetaxel and bortezomib, there was no improved efficacy when compared to prior studies which used docetaxel alone. It was also found that bortezomib has minimal activity in patients with CRPC and is unlikely to make an impact on treatment efficacy (NCT00059631). Another trial focusing on the safety of bortezomib as pre-treatment in those about to undergo prostatectomy found that none of the subjects were affected by poor wound healing or excessive bleeding (NCT00425503).

### 7.5. PI3K/AKT/mTOR Pathway

One clinical trial (NCT01717898) for BEZ235 was terminated due to high toxicity. Another clinical trial (NCT01634061) for BEZ235 was completed and is pending results.

### 7.6. Microenvironment (VEGF)

In a clinical trial evaluating the efficacy of bevacizumab and erlotinib, it was found that 7 of 19 subjects had tumor recurrence after an average of 285 days (NCT00203424). Another study found that the average time to tumor progression was seven months in those taking satraplatin and bevacizumab in mPca patients previously treated with docetaxel (NCT00499694). One study found that in those receiving ADT alone the average relapse free survival rate was 13 months compared to those receiving ADT and bevacizumab, who had an average relapse free survival of 10 months (NCT00776594). Another trial found that, for every one patient, 0.22 patients experienced an endorectal MRI response after completion of six cycles of neoadjuvant therapy of bevacizumab plus docetaxel (NCT00321646). One trial evaluating the PSA and immune response to docetaxel, thalidomide, prednisone, and bevacizumab found that 52 of 60 subjects had a PSA response (NCT00089609). Another trial focused on determining the maximum tolerated dose of temsirolimus with a fixed dose of bevacizumab found that 5 of 16 subjects had a change from baseline PSA at a MTD of 25 mg (NCT01083368). In those with non-metastatic CRPC receiving bevacizumab, it was found that 5 of 15 subjects had a PSA decline of >/=50 percent, 14 of 15 experienced drug toxicities, and the average time to PSA progression was 2.8 months (NCT-1656304). It was also found that 98 percent of subjects had PSA progression at one year in those receiving bevacizumab one year following treatment with ADT (NCT00658697). Those taking docetaxel and placebo survived an average of 21.5 months while those receiving docetaxel plus bevacizumab survived an average of 22.6 months in those who did not respond to ADT (NCT00110214). Those taking gemcitabine hydrochloride, cisplatin, and bevacizumab survived an average of 14.5 months, while those taking the same regimen but without bevacizumab survived 14.3 months (NCT00942331).

### 7.7. CAR-T

A clinical trial (NCT03013712) for EpCAM currently has unknown status.

### 7.8. Nanoparticles

Two clinical trials (NCT01300533 and NCT02646319) have been completed and are pending results. One clinical trial (NCT02769962) is currently recruiting with projected completion in 2023. Two clinical trials (NCT03531827 and NCT04221828) were terminated due to toxicity and lack of enrollment, respectively. One clinical trial (NCT00499291) was withdrawn for unknown reasons.

There are currently 52 clinical trials of interest available on the targeted therapies mentioned. Only 13 have been completed with available results as of 8 January 2023. Furthermore, 16 of the studies mentioned in Table 1 describe only safety and maximum tolerated dose of certain therapies rather than the overall effectiveness of treatment. These studies were included in this review, since the safety of the therapy is as important as the efficacy in most cases.

The aforementioned 13 completed trials involve only three of the different targeted pathways. For the Hh pathway, trials involving GDC-0449 are incomplete, and there are currently no trials involving GD-61. For the Wnt pathway, there are no studies involving 3289–8625, LGK974, or OMP-54F28, and the trials involving Foxy-5 do not have results. The trial involving RO4929097 of the Notch pathway was terminated. Regarding NF-kB, there are no trials with PS1145, BMS345541, or 17-(allylamino)-17-demethoxygeldanamycin, and there are no results for the trials using bortezomib, aspirin, or BKM120. Both of the PI3K/AKT/mTOR pathway trials targeting BEZ235 do not have results. There is one clinical trial looking at EpCAM for CAR-T that has had unknown status since 2017, and no trials involving anti-CD133. The highest number of trials with completed results involved VEGF-targeting bevacizumab therapy, comprising 10 of the 13 completed clinical trials with available results. It should be noted that no clinical trials involving nanoparticle therapy specifically targeting PCSC were able to be found. However, several trials involving more generalized nanoparticle therapy for advanced PCa were included to highlight the current state of investigation.

At this time, 15 clinical trials have been marked as complete but have not posted results, with 9 of these investigating the safety of treatment only. These findings will hopefully provide further insight into the effectiveness of the Wnt-targeting Foxy-5, NF-kB-targeting bortezomib/aspirin/BKM120, and PI3K/AKT/mTOR-targeting BEZ235. Furthermore, the results of the seven currently active trials are also highly anticipated and will provide additional information on the Hh-targeting GDC-0449, NF-kB-targeting bortezomib, and VEGF-targeting bevacizumab therapies.

## 8. Conclusions and Future Directions

In conclusion, based on this review of potential clinical targets and available clinical trials, it is clear that a significant need exists for further research and testing into these targeted therapies. The intricacies and interactions among the developmental pathways identified here still require a great deal of investigation to better understand their targeting effects. Most of the developed therapies still require extensive testing with regard to their feasibility, safety, efficacy, and dosing. Yet, even with the limited results published to date, it remains clear that targeted anti-CSC therapies have the potential for significant clinical impact on the treatment of CRPC and remain an important avenue for future treatment. In agreement with Wolf et. al., future characterization of PCSCs using genomics and proteomics would increase our knowledge of PCSC targets of interest, increase treatment possibilities, and provide more individualized therapy for those diagnosed with prostate cancer even in advanced stages [128].

## Figures and Tables

**Table 1:** A summary of all clinical trials via clinicaltrials.gov (accessed on 15 January 2023) available on the targeted therapies discussed including Hedgehog (Hh) pathway and targeting with inhibitors (sonidegib, *GANT-61*, and GDC-0449), Wnt signaling pathway and targeting with inhibitors (3289–8625, LGK974, Foxy-5, and OMP-54F28), Notch pathway and targeting with inhibitors (RO4929097), NF-κB pathways and targeting with inhibitors (bortezomib, PS1145, BMS345541, aspirin, 17-(allylamino)-17-demethoxygeldanamycin, and BKM120), PI3K/AKT/mTOR pathway and targeting it with inhibitors (BEZ235), prostate CSC microenvironment (Bevacizumab), and immunotherapies (anti-C133 CAR-T therapy and CAR T cells targeting the CSC marker EpCAM).

Pathway	Target	NCT Number	Phase	Status	Notes
**Hh**	Sonidegib	NCT02111187	1	Completed	Primary outcome: change from baseline in tissue GLi1 expression levels in those receiving LDE225, compared to those receiving no treatment prior to prostatectomy. Results: 6/7 participants had at least a 2-fold reduction in GLi1 expression in post treatment vs. pretreatment tumor tissue.
		NCT02182622	1	Withdrawn	Primary outcome: maximum tolerated dose of LDE225 plus docetaxel/prednisone. Withdrawn: no reason reported.
	GDC-0449	NCT01163084	1,2	Terminated	Primary outcome: proportion of patients with </=5% tumor involvement in those receiving leuprolide acetate or goserelin acetate with or without vismodegib. Terminated: no reason reported.
		NCT 00607724	1	Completed	Primary outcome: percentage of participants with dose-limiting toxicities (DLTs) Results: 0/68 subjects affected.
		NCT02465060	2	Recruiting	Primary outcome: objective response rate in those receiving targeted therapy directed by genetic testing. Recruiting: projected completion 2025.
**Wnt**	Foxy-5	NCT02020291	1	Completed	Primary outcome: safety and tolerability of Foxy-5. No results posted.
		NCT02655952	1	Completed	Primary outcome: presence of dose limiting toxicities of Foxy-5. No results posted.
**Notch**	RO4929097	NCT01200810	2	Terminated	Primary outcome: time to PSA progression in those receiving bicalutamide and RO4929097. Terminated: lack of study drugs.
**NF-kB**	Bortezomib	NCT00103376	2	Terminated	Primary outcome: PSA response in those receiving bortezomib with or without hormone therapy. Terminated: low accrual.
		NCT00183937	2	Completed	Primary outcome: number of patients with improved serum PSA response rate in those receiving bortezomib and docetaxel. No results posted.
		NCT00059631	1	Completed	Primary outcome: maximum tolerated dose of mitoxantrone combined with bortezomib. No results posted.
		NCT00193232	2	Completed	Primary outcome: objective response rate. Results: treatment with combination of weekly docetaxel and bortezomib showed no improved efficacy vs. previous results with docetaxel alone, bortezomib has minimal activity in pts with HRPC and is unlikely to make any impact on treatment efficacy.
		NCT00425503	2	Completed	Primary outcome: assess the safety of PS-341 as a pretreatment in patients who are to undergo a radical prostatectomy measured with poor wound healing and excessive bleeding. Results: 0/37 subjects affected.
		NCT00064610	1,2	Completed	Primary outcome: determine the maximum tolerated dose and preliminary activity of PS-341 plus docetaxel. No results posted.
		NCT00667641	1	Completed	Primary outcome: maximum tolerated dose of paclitaxel in combination with bortezomib. No results posted.
		NCT00620295	1	Completed	Primary outcome: maximum tolerated dose of bortezomib and gemcitabine. No results posted.
	Aspirin	NCT03819101	3	Recruiting	Primary outcome: overall survival in those taking aspirin and atorvastatin. Recruiting: projected completion 2034.
		NCT02757365	4	Unknown	Primary outcome: aspirin PSA response, digital rectal exam, US of prostate, biopsy, fPSA level. Unknown: last status verification in 2016
		NCT02420652	2	Terminated	Primary outcome: change in stable PSA rates after 6 months of metformin hydrochloride and aspirin or placebo. Terminated: slow accrual.
		NCT01428869	Unknown	Completed	Primary outcome: diagnosis of prostate cancer in REDUCE study participants treated with statins, aspirin, and dutasteride. No results posted.
		NCT00234299	NA	Completed	Primary outcome: assess the effect of oral aspirin on in vivo prostate epithelial cells. No results posted.
		NCT02804815	3	Recruiting	Primary outcome: survival and disease recurrence in those receiving aspirin after primary therapy. Recruiting: projected completion 2026.
	BKM120	NCT01695473	2	Terminated	Primary outcome: percent of patients with decrease in phosphorylated S6 ICH from baseline, percent with downstream target inhibition of PI3K in prostate tumor tissue measured by ICH when treated with BKM120. Terminated: lack of accrual.
		NCT01634061	1	Completed	Primary outcome: incidence of dose limiting toxicities and PSA decline >/= 30% in those getting abiraterone acetate and BEZ235 or BKM120. No results posted.
		NCT01385293	2	Terminated	Primary outcome: progression free survival prostate cancer working group 2 criteria or based on the onset of a skeletal related event in those getting BKM120. Terminated: 1st stage due to futility
		NCT02035124	2	Withdrawn	Primary outcome: number of subjects with serious and non-serious adverse events and progression free survival in those receiving cabazitaxel and BKM120. Withdrawn: slow accrual, no subjects enrolled.
		NCT02487823	1	Terminated	Primary outcome: determine MTD of BKM120 when given orally in combination with daily bicalutamide and LH-RH agonists. Terminated: defect of recruitment.
		NCT01741753	1	Terminated	Primary outcome: safety profile and mTD for BKM120/abiraterone/prednisone. Terminated: slow accrual, supplier of BKM120 asked to cease further enrollment.
**PI3K/AKT/mTOR**	BEZ235	NCT01717898	1,2	Terminated	Primary outcome: number of reported DLT and MTD when combining BEZ235 and abiraterone acetate, decline in PSA > 50%. Terminated: DLT on lowest dose level.
		NCT01634061	1	Completed	Primary outcome: incidence of DLT and PSA decline >/= 30% in those getting abiraterone acetate and BEZ235 or BKM120. No results posted.
**VEGF**	Bevacizumab	NCT00203424	2	Completed	Primary outcome: evaluate efficacy of bevacizumab and erlotinib, time to tumor recurrence. Results: 7/19 subjects had tumor recurrence, average 285 days.
		NCT00499694	NA	Completed	Primary outcome: time to progression in those taking satraplatin and bevacizumab in mPCa patients previously treated with docetaxel. Results: average time to progression 7 months.
		NCT00574769	1,2	Completed	Primary outcome: MTD of RAD001 with docetaxel/bevacizumab. No results posted.
		NCT00776594	2	Completed	Primary outcome: relapse-free survival in those treated with ADT vs. ADT plus bevacizumab. Results: average relapse free survival of 13 months vs. 10 months respectively.
		NCT00321646	2	Completed	Primary outcome: endorectal MRI response after completion of 6 cycles of neoadjuvant therapy in subjects receiving bevacizumab plus docetaxel. Results: 22% of participants had a response.
		NCT00027599	2,3	Completed	Primary outcome: efficacy of APC8015 and bevacizumab in terms of decline in PSA and effect on PSA doubling time. No results posted.
		NCT00348998	2	Unknown	Primary outcome: determine the safety and efficacy of bevacizumab with hormonal therapy and radiotherapy. Unknown: last status update in 2008 stated active, not recruiting.
		NCT00089609	2	Completed	Primary outcome: number of participants with PSA response and immune response to docetaxel, thalidomide, prednisone, and bevacizumab. Results: 52/60 subjects had PSA response, immune response not measured.
		NCT01083368	1,2	Completed	Primary outcome: MTD of temsirolimus with fixed dose bevacizumab and objective response via PSA level. Results: MTD of temsirolimus was 25 mg, 5/16 subjects had a change from baseline PSA.
		NCT01656304	2	Completed	Primary outcome: PSA response rate with bevacizumab in non-metastatic CRPC, toxicities of bevacizumab, and time to PSA progression. Results: 5/15 subjects had a PSA decline >/=50%, 14/15 subjects had toxicities, time to PSA progression = average 2.8 months.
		NCT00016107	2	Completed	Primary outcome: time to objective progression, objective and PSA response rates, and toxicity in those receiving chemo plus bevacizumab. No results posted.
		NCT00658697	2	Completed	Primary outcome: PSA progression at 1 year after completing ADT. Results: 98% had PSA progression at 1 year.
		NCT00110214	3	Completed	Primary outcome: overall survival in those receiving docetaxel and prednisone with or without bevacizumab who did not respond to hormone therapy. Results: those taking docetaxel plus placebo survived average 21.5 months, docetaxel plus bevacizumab survived average 22.6 months.
		NCT00942331	3	Completed	Primary outcome: overall survival in those receiving gemcitabine hydrochloride and cisplatin with or without bevacizumab. Results: those taking gemcitabine hydrochloride, cisplatin, and bevacizumab survived average 14.5 months, gemcitabine hydrochloride, cisplatin, and placebo survived average 14.3 months.
		NCT05489211	2	Recruiting	Primary outcome: objective response rate, number of subjects with adverse events in those receiving dato-dxd monotherapy and in combination with anti-cancer drugs including bevacizumab. Recruiting: projected completion 2025.
**CAR-T**	EpCAM	NCT03013712	1,2	Unknown	Primary outcome: toxicity profile of EpCAM targeted CAR T cells with CTCAE. Unknown: last status update 2017 stated recruiting.
**Nanoparticles**	Nanoparticle-Based Drug Delivery System	NCT00499291	NA	Withdrawn	Primary outcome: develop a population pharmacokinetic model in those receiving paclitaxel albumin-stabilized nanoparticle formulation. Withdrawn: no reason reported
		NCT04221828	2	Terminated	Primary outcome: number of adverse events in those receiving NanoPac (sterile nanoparticle paclitaxel). Terminated: lack of enrollment
		NCT03531827	2	Terminated	Primary outcome: percentage of participants with anti-tumor activity with combined CRLX101 and enzalutamide in those who previously failed enzalutamide therapy. Terminated: closed due to toxicity
		NCT02769962	2	Recruiting	Primary outcome: determine overall response rate of EP005 plus Olaparib in mCRPC. Results: projected completion 2023
		NCT02646319	1	Completed	Primary outcome: clinical benefit rate, incidence of adverse events, survival time, and time to disease progression in those receiving nanoparticle albumin-bound rapamycin. No results posted.
		NCT01300533	1	Completed	Primary outcome: determine DLT of BIND-014. No results posted.
